# Fanconi anemia proteins counteract the implementation of the oncogene-induced senescence program

**DOI:** 10.1038/s41598-019-53502-w

**Published:** 2019-11-19

**Authors:** Anne Helbling-Leclerc, Françoise Dessarps-Freichey, Caroline Evrard, Filippo Rosselli

**Affiliations:** 1 UMR8200-CNRS, Gustave Roussy, Villejuif, Cedex France; 2 Université Paris-Sud, Université Paris-Saclay, Orsay, France; 3 Equipe labellisée “La Ligue Contre le Cancer”, Villejuif, France

**Keywords:** Oncogenes, Senescence

## Abstract

Fanconi Anemia (FA), due to the loss-of-function of the proteins that constitute the FANC pathway involved in DNA replication and genetic stability maintainance, is a rare genetic disease featuring bone marrow failure, developmental abnormalities and cancer predisposition. Similar clinical stigmas have also been associated with alterations in the senescence program, which is activated in physiological or stress situations, including the unscheduled, chronic, activation of an oncogene (oncogene induced senescence, OIS). Here, we wanted to determine the crosstalk, if any, between the FANC pathway and the OIS process. OIS was analyzed in two known cellular models, IMR90-hTERT/ER:RAS^G12V^ and WI38-hTERT/ER:GFP:RAF1, harboring 4-hydroxytamoxifen-inducible oncogenes. We observed that oncogene activation induces a transitory increase of both FANCA and FANCD2 as well as FANCD2 monoubiquitination, readout of FANC pathway activation, followed by their degradation. FANCD2 depletion, which leads to a pre-senescent phenotype, anticipates OIS progression. Coherently, FANCD2 overexpression or inhibition of its proteosomal-dependent degradation slightly delays OIS progression. The pro-senescence protease cathepsin L, which activation is anticipated during OIS in FANCD2-depleted cells, also participates to FANCD2 degradation. Our results demonstrate that oncogene activation is first associated with FANCD2 induction and activation, which may support initial cell proliferation, followed by its degradation/downregulation when OIS proceeds.

## Introduction

Cellular senescence defines a genetically controlled physiological program which activation leads to permanently growth-arrested cells that are unable to resume replication^1–4^. Three types of senescence have been described^5,6^: replicative senescence, originally reported by Hayflick, which is triggered by telomere attrition as a consequence of the progressive accumulation of replication cycles^7^, stress-induced senescence, as in the presence of unrepairable DNA damage^8^, and oncogene-induced senescence (OIS), which is activated in response to the aberrant expression of an oncogene^9–11^. Cellular senescence is not simply a program associated with organism aging or a way to limit cell death and tissue disorganization by maintaining living metabolizing cells. Indeed, senescence is also a key physiological mechanism involved in embryonic development, where it appears to be regulated by the TGFβ-SMAD and the PI3K-FOXO signaling pathways^12^. Importantly, senescent cells retain some metabolic activities, including the capability to secrete pro-inflammatory cytokines and reactive oxygen species (ROS) and to maintain recycling networks that provide metabolites and nutrients necessary to sustain themselves^3,4^.

The senescence program induced in response to the unrestrained activation of an oncogene is considered an anticancer barrier that prevents initiated cells from progressing to cancer^9–11,13,14^. A situation exemplified by the human nevi containing melanocytes that, despite the presence in their genome of oncogene activating mutations, may remain for decades in a non-proliferating status before their eventual awakening and progression to melanoma^15^. Although it is not clear how an oncogene-induced senescent cell may by-pass the established senescent program and re-enter in proliferation, proteins involved in DNA damage response (DDR) appear to have a role in both senescent establishment and escaping^16–19^.

We recently reported that the FA core complex and its target FANCD2, which loss-of-function is associated with the hematopoietic, developmental and cancer predisposition disease Fanconi anemia (FA)^20,21^, protects metastatic melanoma cell lines bearing the BRAF^V600E^ mutation and overexpressing MiTF from entering senescence, sustaining melanoma proliferation *in vitro* and *in vivo*^22^. The FANC proteins and their numerous partners inside the so-called FANC pathway constitute a major genetic stability maintenance network involved in DNA repair and replication rescue^20,21,23^. Although the analysis of telomere attrition in FA generated conflicting results, the Hayflick limit of FA primary fibroblasts in standard laboratory culture conditions is significantly reduced compared to that of WT cells, supporting a role of the FANC pathway in replication- and/or DNA damage-induced senescence^22,24,25^. Moreover, FANC pathway loss-of-function was associated with the induction of several genes/proteins that negatively control cell cycle progression, including p53, p21 and p16, with the cellular accumulation of reactive oxygen species (ROS) and with alterations in both TGFβ-SMAD and PI3K-FOXO pathways^22,26–28^. All the previous features, together with the induction of senescence-associated β-galactosidase activity, the establishment of new histone marks landscape and the accumulation of DAPI-dense senescence associated heterochromatin foci (SAHF) constitute major hallmarks of a senescent phenotype^2^. Thus, the previous observations support a general anti-senescent role of the FANC pathway, and loss of signaling through this pathway likely constitutes a major event in the development of the disease. However, little is known about how the FANC pathway regulates senescence.

In this study, mainly by using FANCD2 as readout, we wanted shed light on the role, if any, and the behavior of the FANC pathway in the context of the OIS program. By analyzing two *in vitro* models in which OIS could be activated in a controlled manner, we present robust evidence that FANCD2 is activated at the beginning of the process and successively degradated allowing senescence to progress robustly.

## Results

### Oncogene-induced senescence is associated to a rapid induction of FANCD2 expression and monoubiquitination followed by its downregulation

**Figure 1:**
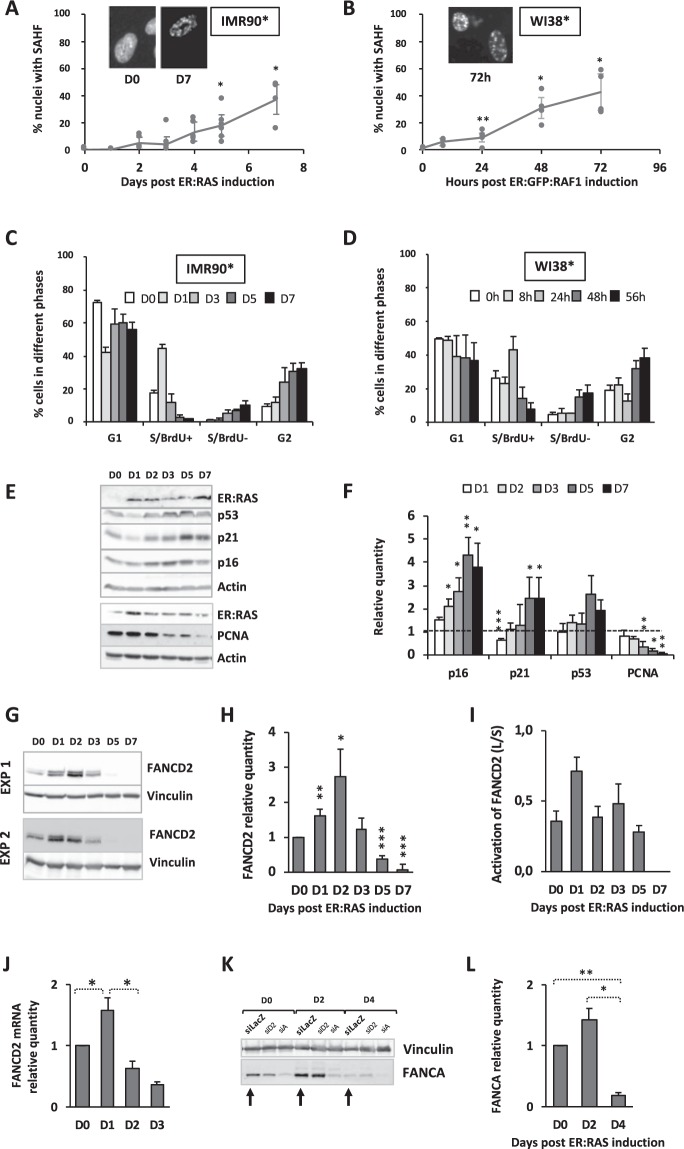
OIS is associated to a rapid raise in FANCD2 expression and monoubiquitination followed by its downregulation. **(A,B)** Frequency of SAHF-positive cells in IMR90-hTERT/ER:RAS (IMR90*) and WI38-hTERT/ER:GFP:RAF1 (Wi38*) cells exposed to 4HT (100 nM and 20 nM, respectively) during the indicated times to induce and maintain oncogene expression. The morphological changes in nuclear structure are shown after DAPI staining. Each point represents the mean +/− SEM of at least 3 independent experiments. *p < 0.05; **p < 0.01. **(C,D)** Cell cycle profile analysis at the indicated time points following oncogene induction in IMR90* and WI38* cells. Bars represent the mean +/− SEM of three independent experiments. See Fig. S1A,B for FACS analysis. **(E)** Representative Western blots showing the expression of the senescence-associated markers p16, p21, p53 as well as the S-phase specific protein PCNA in IMR90* cells. Actin is used as loading control. **(F)** Quantitative analysis of the p16, p21, p53 and PCNA expression at the indicated times in oncogene-activated IMR90* cells compared to their level in non-activated cells. Bars represent the mean +/− SEM of at least 3 independent experiments. *p < 0.05; **p < 0.01; ***p < 0.005. **(G)** Western blots from two experiments showing the time course expression and activation of FANCD2 in IMR90* cells with an activated-RAS^v12^ oncogene. Vinculin is used as loading control. **(H)** Quantitative analysis of FANCD2 expression at the indicated time point in oncogene-activated IMR90* cells. Bars represent the mean +/− SEM of at least 3 independent experiments. *p < 0.05; **p < 0.01; ***p < 0.001. **(I)** Quantitative analysis of FANCD2 activation, i.e., ratio L/S, monoubiquitinated/non-monoubiquitinated form of FANCD2, at the indicated time point in oncogene-activated IMR90* cells. **(J)** Quantitative analysis of FANCD2 mRNA at the indicated time points in oncogene-activated IMR90* cells. Bars represent the mean +/− SEM of 4 independent experiments. *p < 0.05. **(K)** Representative Western blots showing the time course expression of FANCA in IMR90* cells with an activated-RAS oncogene. Control cells transfected with siLacZ were indicated by arrows. Vinculin is used as loading control. **(L)** Quantitative analysis of FANCA expression at the indicated time points in oncogene-activated IMR90* cells. Bars represent the mean +/− SEM of at least 3 independent experiments. *p < 0.05; **p < 0.01.

To determine the involvement of the FA pathway in the establishment of the OIS program, we used two well-known and widely used cellular systems: the IMR90-hTERT fibroblasts bearing an ER:RAS^G12V^ ^29^ and the WI38-hTERT/ER:GFP:RAF1^30^ (noted, respectively, IMR90* and WI38* from now onwards and in figures), in which oncogene expression is obtained by 4-hydroxytamoxifen (4HT) exposure. The two systems differ in the rapidity of the accumulation of SAHF positive cells, which we used as cellular marker of established senescence following oncogene induction (Fig. 1A,B). Indeed, RAS expression in IMR90* cells led to approximately 40% of cells presenting nuclear SAHF one week after 4HT exposure, whereas a WI38* cell population expressing RAF1 reached a similar level of senescent cells in around 72 h. In both models, oncogene activation is followed by a wave of replication within 24 h before undergoing growth arrest in both G1 and G2 (Fig. 1C,D and Supplemental Fig. S1A,B), suggesting that oncogene activation leads to a “go” signal, which pushes G1-licensed cells to a rapid entry in replication followed by a “stop” signal that “freezes” the cells in G1 and G2, impeding proliferation. Accordingly, the intracellular level of several key growth arrest mediators, including p53, p16 and p21, increased following oncogene activation whereas that of PCNA, a S-phase marker, decreased (Fig. 1E,F, for IMR90*, and Supplemental Fig. S2A,B for WI38*).

Activation of the OIS program was associated with a notable increase in FANCD2 expression, at both mRNA and protein level, and monoubiquitination, readout of an activated FANC core complex (Fig. 1G–I for IMR90* and Supplemental Fig. S2A,C for WI38*). Accordingly, with its roles in safeguarding S phase progression, FANCD2 expression and activation appear associated with the wave of S-phase, BrdU-incorporating, cells. FANCD2 expression was completely lost five days or 72 h following oncogene activation, respectively, in IMR90* and WI38* (Fig. 1G–I for IMR90* and Fig. S2A,C for WI38*), when the cells stop to proliferate. FANCD2 downregulation during OIS progression was associated to a decay in its mRNA level (Fig. 1J). Finally, the expression of the FA core complex partner FANCA was also rapidly but transitorily increased following oncogene activation (Fig. 1K,L).

Altogether, our data demonstrate that OIS induction is associated to FANC pathway activation concomitantly to the raise in S-phase, BrdU incorporating cells before the induction of p53, p16 and p21, molecular events likely involved in the successive growth arrest in G1 and G2.

### FANCD2 participates to cellular homeostasis, counteracting senescence during normal proliferation as well as following oncogene activation

The previous results raise the question of whether the behavior of the FANC proteins during OIS progression is an integral part of the senescence program or a simply epiphenomenon associated to the replicative activity of the cells. Thus, to understand the role of the FANC pathway in the senescence program, we decided to monitore OIS progression in cells either depleted for FANCD2 or overexpressing it.

**Figure 2:**
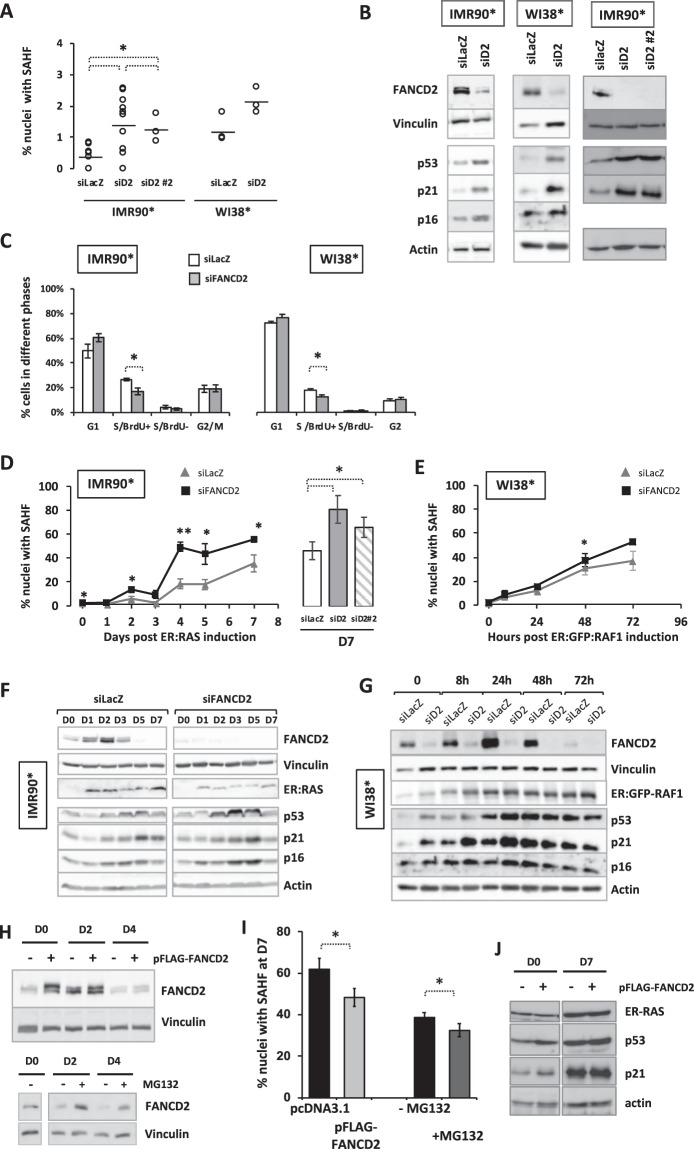
FANCD2 couteracts OIS. **(A)** Frequency of SAHF-positive cells in IMR90* or WI38* transfected with an untargeted siRNA or with siRNAs (siD2 or siD2 #2) targeting FANCD2. Each point represents an independent experiment. Horizontal traits indicated the calculated mean. *p < 0.05. **(B)** Representative Western blots showing the expression of indicated proteins in siRNA-depleted FANCD2 IMR90* or WI38* cells. Vinculin and actin are used as loading control. Two different siRNA targeting FANCD2 (siD2 or siD2 #2) were tested in IMR90*. **(C)** Cell cycle profiles in IMR90* or WI38* transfected with an untargeted siRNA or with siRNA targeting FANCD2. The percentage of cells in each phase was evaluated by FACS analysis. Bars represent the mean +/− SEM of 3 independent experiments. *p < 0.05. **(D,E)** Time-course accumulation of SAHF-positive cells in FANCD2-proficient or FANCD2-depleted IMR90* (**D**) or WI38* (**E**) cells. Histogram represents comparison of SAHF frequency at day 7 obtained in two different FANCD2-depleted IMR90* cells. Each point represents the mean +/− SEM of at least 3 independent experiments. *p < 0.05; **p < 0.01. **(F,G)** Representative Western blots showing the time-dependent expression of the indicated proteins in FANCD2-proficient or FANCD2-depleted IMR90* (**F**) or WI38*cells (**G**) expressing their specific oncogene. See Fig. S2D for quantification. Vinculin and actin are used as loading control. **(H**) Top. Representative Western blots showing the time-dependent expression of FANCD2 in pFLAG-FANCD2 transfected IMR90* cells or control cells transfected with empty plasmid (pcDNA3.1). Bottom. Untransfected cell treated with the proteasome inhibitor MG132 (2 µM, overnight). Vinculin is used as loading control. **(I)** Frequency of SAHF-positive cells after 7 days of oncogene induction in (Left) pFLAG-FANCD2 transfected IMR90* cells or control cells transfected with empty plasmid (pcDNA3.1) or (right) cell treated with the proteasome inhibitor MG132 (2 µM, overnight before fixation). Data represent the mean +/− SEM of at least 3 independent experiments. *p < 0.05. **(J)** Representative Western blot showing the time-dependent expression of the indicated proteins in pFLAG-FANCD2-transfected cells or control transfected cells. Actin is used as loading control.

First, we analyzed the consequences of the siRNA-mediated downregulation of FANCD2 on IMR90* and WI38* cells looking at the accumulation of SAHF-positive cells, their cell cycle profile, and the expression of p53, p21 and p16 in the absence of oncogene activation. FANCD2 depletion is followed by a slight but consistent increase in the percentage of cells presenting SAHF and in the levels of p53, p21 and p16 (Fig. 2A,B) whereas the frequency of BrdU-incorporating cells slightly diminishes (Fig. 2C). Targeting FANCD2 with a different siRNA gave similar results (Fig. 2A,B). Thus, a FANC pathway-deficient cell population at steady state presents all the key hallmarks of senescence. This finding supports two, non-mutually exclusive, possibilities: either a physiological function of the FANC pathway is to counteract the activation of the senescence program or the increased level of DNA damage, known to be associated to FANC pathway loss-of-function, constitutes a pro-senescence signal.

Successively, we analyzed the behavior of FANCD2-depleted IMR90* or WI38* cells following oncogene induction. As particularly evident in IMR90* cells, FANCD2 depletion significantly increased the frequency of SAHF-positive cells (or anticipated their accumulation) in response to an oncogenic signal without major modifications in the levels of p53, p21 and p16 (Fig. 2D–G and Supplemental Fig. S2D), suggesting that they reach the necessary threshold allowing senescence progression and maintenance independently of the FANC pathway activity.

Next, we transfected IMR90* cells with a vector expressing a FLAG-FANCD2 construct, induced RAS expression 48 h later and followed the behavior of cells. Even if plasmid transfection *per se* increased the frequency of senescent cells, FANCD2 overexpression (Fig. 2H top) was able to slightly reduce the percentage of SAHF-positive cells at 7 days post-oncogene expression (Fig. 2I), without major effect on p53 or p21 induction (Fig. 2J). Notably, compared to the clear difference observed when the oncogene expression was induced (D0) the level of FANCD2 4 days (4D) later is similar in both mock-transfected and overexpressing cells (Fig. 2H). The previous observation is particularly relevant since it supports that FANCD2 expression is regulated not only at mRNA level (Fig. 1J) but also depends on the degradation of the protein and it also fournishes a possible reason for why the ectopic (over)expression of FANCD2 has a so limited, yet reproducible, effect on SAHF accumulation over the time. Thus, we treated RAS-induced cells with the proteosomal inhibitor MG132, which delaying FANCD2 degradation (Fig. 2H bottom) also slightly delays OIS progression (Fig. 2I).

Altogether, our data suggest that optimal OIS progression is associated to FANCD2 downregulation.

### Higher ROS in FANCD2-deprived cells does not mediate increased SAHF

**Figure 3:**
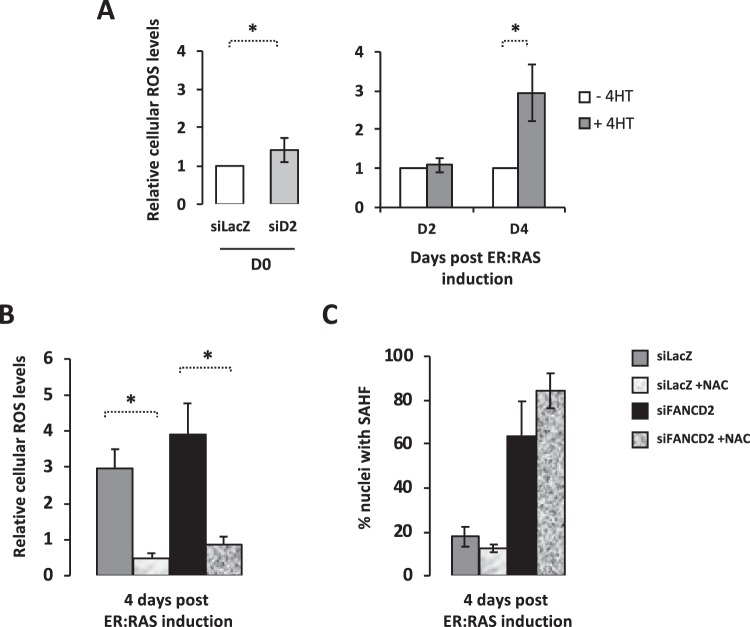
Higher ROS in FANCD2-deprived cells does not mediate increased SAHF. **(A)** Histogram presenting the relative intracellular ROS level in FANCD2-proficient and FANCD2-deficient IMR90* cells before oncogene activation (left) and 2 or 4 days after oncogene activation in control cells (right). Bars represent the mean +/− SEM of 3 independent experiments. *p < 0.05. **(B)** Histogram presenting the relative intracellular ROS level in FANCD2-proficient and FANCD2-deficient IMR90* cells 4 days after oncogene activation in the absence or presence of the NAC (2.5 mM). Bars represent the mean +/− SEM of 3 independent experiments. *p < 0.05. **(C)** Histogram presenting the frequency of SAHF-positive cells in FANCD2-proficient and FANCD2-deficient IMR90* cells 4 days after oncogene activation in the absence or presence of the NAC (2.5 mM). Bars represent the mean +/− SEM of 3 independent experiments.

An increase in intracellular ROS was associated with both OIS and FA pathway deficiency (Fig. 3A). Thus, we sought to determine if and how ROS impact on SAHF formation in FANCD2-proficient and deficient cells. In both oncogene-activated FANCD2-proficient and -deficient IMR90* cells, exposure to the anti-oxidant agent N-acetyl-cysteine (NAC) significantly reduces intracellular ROS levels (Fig. 3B). However, NAC treatment failed to modify the frequency of SAHF-presenting cells independently from FANCD2 expression (Fig. 3C).

Thus, even if FANCD2 is undoubtedly involved in the control of the cellular redox state, our data clearly suggest that FANCD2 affect OIS progression independently of its role in the maintenance of the redox homeostasis.

### Downstream oncogene activation, the senescence program is regulated by a FANCD2/Cathepsin L crosstalk

Previous works established that senescence implementation requires the proteolytic degradation of several DNA repair proteins, including the cathepsin L1 (CTSL1)-mediated degradation of BRCA1 and RAD51, belonging to the FANC pathway downstream FANCD2, and 53BP1^31,32^, key proteins involved in homologous recombination (HR) and non-homologous end-joining (NHEJ).

**Figure 4:**
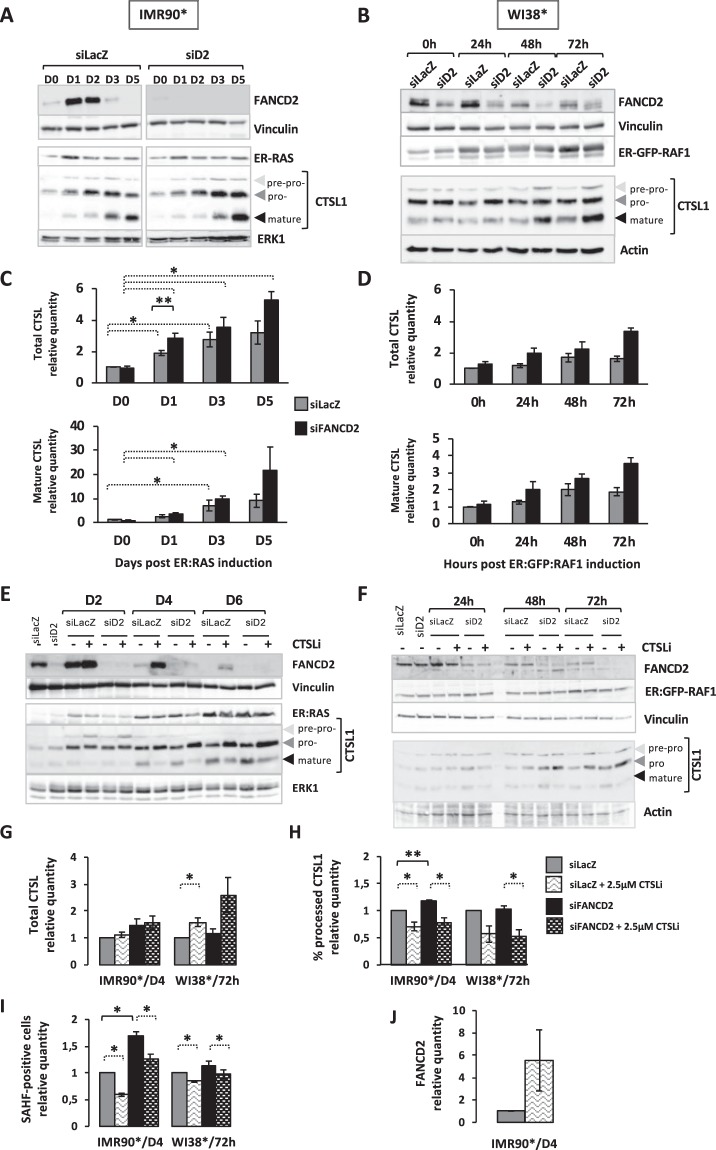
FANCD2 depletion increased cathepsin L1 expression during OIS, a protease targeting FANCD2 downstream oncogene induction. **(A,B)** Representative Western blots showing cathepsin L (CTSL1) behavior in IMR90* (**A**) or WI38* (**B**) cells depleted or not depleted of FANCD2. The three forms of CTSL1 could be observed. ERK1, actin or vinculin were used as loading control. **(C,D)** Histograms representing the time-dependent relative levels of total and mature CTSL1 in oncogene-active IMR90* (**C**) or WI38* (D) cells expressing or not expressing FANCD2. Bars indicate the mean +/− SEM of at least 3 independent experiments. *p < 0.05; **p < 0.01. **(E,F)** Representative Western blot analysis of cathepsin L (CTSL1) and FANCD2 in IMR90* (**E**) or WI38* (**F**) cells depleted or not depleted for FANCD2. Cells were exposed to CTSL1 inhibitor (CTSLi, Z-FF-FMK, dose 2.5 µM) during the OIS kinetic. The three forms of CTSL1 could be observed. ERK1, actin and vinculin were used as loading controls. **(G,H)** Analysis of CTSL1 expression and activation (revealed by its processing) in IMR90* or WI38* cells depleted or not depleted of FANCD2 in absence or presence of a CTSL1 inhibitor. The data were shown as a fold change compared with the value obtained for siLacZ transfected cells. Bars represent the mean +/− SEM of at least 3 independent experiments. *p < 0.05; **p < 0.01. **(I)** Analysis of SAHF-positive cells in IMR90* or WI38* cells depleted or not depleted of FANCD2 in the absence or presence of a CTSL1 inhibitor. The data were shown as a fold change compared with the value obtained for siLacZ transfected cells. Bars represent the mean +/− SEM of at least 3 independent experiments. *p < 0.05. **(J)** Quantitative analysis of FANCD2 expression in IMR90* cells in the absence or presence of a CTSL1 inhibitor. Bars represent the mean +/− SEM of at least 3 independent experiments.

Oncogene activation in IMR90* or WI38* cells is rapidly followed by a progressive increase in both CTSL1 pro- and mature forms (Fig. 4A–D). The siRNA-mediated FANCD2 depletion did not modify *per se* the basal CTSL1 expression and activation. Accordingly, the levels of 53BP1 and RAD51 are known to be unmodified in FANC-deficient cells^33,34^. Oncogene activation in FANCD2-depleted cells is clearly associated to an anticipated activation of CTSL1, parallel to the accelerated rise in SAHF-positive cells observed following FANCD2 depletion (Fig. 4A–D). However, FANCD2 over-expression was unable to modify CTSL1 activation (data not shown). Next, we impeded CTSL1 activition by treating cells with its specific inhibitor Z-FF-FMK (noted CTSLi in Fig. 4E–I) that hamper the conversion of the CTSL1 pro-form in the mature polypeptide, resulting in the accumulation of the first at the expense of the second (Fig. 4E–H). In light of its role in OIS establishment and progression, CTSL1 inhibition reduced the frequency of SAHF-positive cells in FANCD2-proficient, in FANCD2-deficient and in FANCD2-overexpressing cells, stressing the importance of the activated protease in senescence program (Fig. 4I and data not shown). Accordingly with a delayed senescence, CTSL1 inhibition was associated with an increased level of FANCD2 (Fig. 4J), suggesting that the FANCD2-downregulation in oncogene-activated cells is somehow dependent on CTSL1 activity.

Thus, whereas FANCD2 behavior, similarly to that of BRCA1, RAD51 and 53BP1^31,32^, could depend on CTSL1, which activation is required for the optimal progression of the OIS program, CTSL1 expression/activition is FANC-pathway independent.

## Discussion

Fanconi anemia patients present bone marrow failure, developmental abnormalities, reproductive and endocrine defects and cancer predisposition to both solid tumors and leukemia. Patient’s cells are characterized by several cellular phenotypes, including DNA interstrand crosslinking drug sensitivity, chromosome instability, cell cycle abnormalities, oxygen sensitivity and excessive pro-inflammatory cytokines production^20,21^. The proteins encoded by the, up until now, 22 identified genes are major components of the FANC pathway, in which the canonical function is to cope with replication stress^20,21,23^. Their deficiency underlies the genetic instability of the FA patients, their bone marrow failure and their cancer predisposition. However, emerging evidence indicates that certain FANC proteins have alternative functions^21,35–38^, including control of replisome function^24^, modulation of TNF-α expression^39–41^, regulation of the cellular redox equilibrium^26,28^, telomere maintenance^25^ and involvement in virophagy and mitophagy^35^.

In this study, we have shown that an unscheduled and constitutive oncogene activation is followed by a rapid rise in FANCA and FANCD2 at both RNA and protein levels and by FANCD2 monoubiquitination, readout of the pathway activation. However, shortly after the initial increase, FANCD2 expression decreases rapidly as a consequence of a reduced transcription and an increased degradation of the protein. Oncogene activation in siRNA-mediated FANCD2-depleted cells anticipates OIS progression. Conversely, overexpression of FANCD2 or inhibition of its degradation (Figs 2 and 4) delays OIS progression. The previous observations suggest that FANC pathway is initially activated to timely respond to a proliferation signal to sustain replication and cell division, its known canonical key function. Nevertheless, when the expression of an oncogene (a proliferation signal) is constitutively maintained leading to a prolonged “stressed” replication that with associated DNA damage activates checkpoints resulting in the progression of the senescence program, which shortens FANC protein expression at RNA level, as a consequence of growth arrest and at protein level downstream proteasome and CTSL1 activation. These results validate and extend our previous data obtained on melanoma cells in which FANC pathway loss-of-function was rapidly followed by the cellular entry into senescence^22^. While it was demonstrated that the FA core complex and FANCD2 are negatively involved in the control of the cellular redox state by their biochemical and functional interactions with redox-controlling enzymes and proteins, our data clearly suggest that FANCD2 counteracts SAHF-positive cell accumulation independently of its role in the maintenance of the redox homeostasis. Furthermore, we demonstrated that the rapid FANCD2-downregulation in oncogene-activated cells is also partially dependent on the protease CTSL1, known to be responsible for the proteolysis of several other proteins, including BRCA1 and RAD51, which belong to the FANC pathway, and 53PB1, which unscheduled utilization in FANCD2- or BRCA1-deficient cells leads to complex chromosome aberrations, including radial figures^34^. Thus, even if ectopically overexpressed, FANCD2 is actively degradated in oncogene induced cell, supporting the reduced, but still significant, decrease of SAHF frequency observed in pFLAG-FANCD2 transfected cells. Notably, as previously reported when BRCA1 is downregulated, we observed an anticipation of the CTSL1 activation downstream oncogene activation^32^.

**Figure 5:**
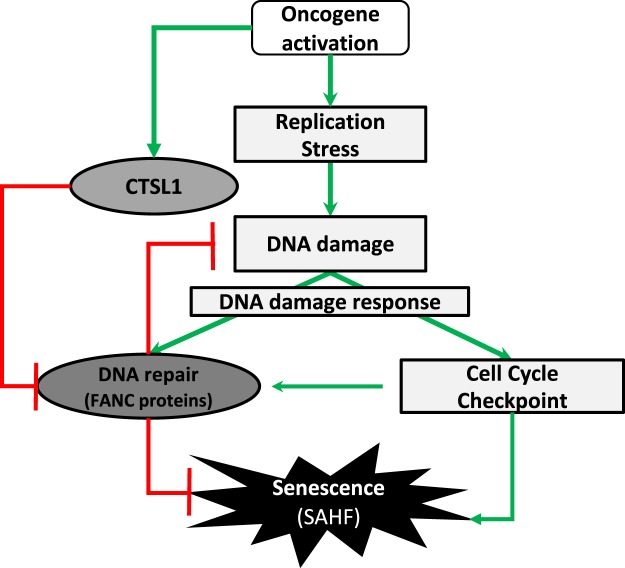
Model presenting the interplay between oncogene activation, FANCD2 and cathepsin expression. An unscheduled and permanent oncogene activation leads to a burst of cells in S-phase associated to DNA damage and replication stress that activates the DNA damage response. FANCD2, which activation is integrated in the DDR network, acts to not only allow DNA repair and replication rescue but also delay the potential establishment of a permanent growth arrest signaling network that drives the cells into senescence by opposing to CTSL1 activity that, *per se*, affects, directly or indirectly, FANCD2 expression.

In conclusion, together with published observations, our data lead to propose a simplified model presented in Fig. 5. Unscheduled and permanent oncogene activation leads to a burst of cells in S-phase, followed by a permanent growth arrest state associated with DDR and CTSL1 activation. The DDR network is constituted by two interconnected branches: the checkpoint pathways and the DNA repair pathways, with the first being essential to the activation of the second. Indeed, by forcing cells to enter into S and by modifying the replication physiology, oncogene expression activates cell cycle checkpoints, including ATM/ATR, p53, p16, p21, CHK1 and CHK2, which participate to the growth arrest, and DNA repair pathways, including FANCA, FANCD2, BRCA1 and RAD51 which work as partners in the FANC pathway, triyng to eliminate induced genomic stresses to allow cell cycle rescue. When the proliferation signal induced by an activated oncogene is not turned off, catabolic pathways are activated, as here CTSL1, to stop futile DNA repair activities, to permanently arrest cell proliferation and to push cells into senescence. Notably, the FANC pathway, embedded in the DDR network, acts to not only allow replication rescue but also to relieve the growth arrest signaling that drives the cells into senescence^42^.

Despite the premature senescence observed in FANCD2 depleted cells, cancer predisposition is observed in FA. This finding could be explained by the “dark-side” of senescence. Indeed, FA cells are genetically unstable, and the senescent cells and their SASP (senescent-associated secretary phenotype) can act extrinsically to promote neoplastic transformation of premalignant cells^43^ that escape senescence to become tumoral.

## Methods

### Cell culture

IMR90-hTERT/ER:RAS^G12V^ cells (immortalized human embryonic lung fibroblasts) were immortalized by C. Mann (CEA/Saclay, France) by introduction of the hTERT gene into IMR90-ER:RAS^G12V^ cells provided by N. Masashi^29^. The cells were grown in DMEM + 15% FBS + 2 mM of L-glutamine + 1 mM of pyruvate + 0.1 mM of non-essential amino acids + 100 µg/mL streptomycin + 100 U/mL penicillin (Gibco).

WI38-hTERT/ER:GFP:RAF1 cells (immortalized human embryonic fibroblasts) were elaborated by C. Mann^29^. They were grown in MEM-GlutaMAX + 10% FBS + 2 mM L-glutamine + 1 mM of pyruvate + 100 µg/mL streptomycin + 100 U/mL penicillin + 0.2% Fungizone (Gibco).

Cells were cultured in a 5% carbon dioxide and 3% oxygen incubator. Induction of a constitutively active form of RAS fused to the estrogen-receptor binding domain (ER:RAS) or RAF1 fused to GFP and the estrogen-receptor binding domain (ER:GFP:RAF1) was achieved by the addition of 100 nM or 20 nM of 4-hydroxytamoxifen (4HT, Sigma-Aldrich) respectively, and the medium was changed every two days.

### SAHF detection

Cells grown on glass coverslips previously incubated with poly-L-lysine (Sigma-Aldrich) were fixed in 4% paraformaldehyde for 10 min at room temperature prior to permeabilization in 0.1% Triton X-100 for 10 min at RT. The slides were mounted in DAKO mounting medium supplemented with DAPI (1.5 µg/mL, Sigma-Aldrich) and were examined at a magnification of 63X via fluorescence microscopy (AxioImager.Z1, Zeiss). The images were captured with an ORCA-ER camera (Hamamatsu). The microscope and camera parameters were adjusted for each series of experiments to avoid signal saturation.

### Cell cycle analysis

In culture media, 5-bromo-2-deoxyuridine (BrdU, BD Bioscience) was added at a final concentration of 10 μM for 10 min at 37°C. After fixation in iced absolute ethanol, pelleted cells were denatured in 0.5 mg/mL pepsin + 30 mM of HCl. BrdU was immunodetected with a mouse anti-BrdU antibody at 1/50 (DAKO, clone Bu20a) and a fluorescein-conjugated donkey anti-mouse antibody at 1/50 (Life Technologies). Cells were stained with propidium iodide (PI, 25 μg/mL) containing RNase (50 μg/mL) and were analyzed using a FACS (Accuri C6 Flow Cytometer, BD Biosciences).

### Cellular ROS detection

Cells in 6-well dishes were incubated for 30 min at 37°C with 10 μM of carboxy-H_2_DCFDA (Molecular Probe) for IMR90-hTERT/ER:RAS or 5 µM of DHE (Molecular Probe) for WI38-hTERT/ER:GFP:RAF1. The cells were washed with PBS, detached using trypsin and analyzed via FACS (Accuri C6 Flow Cytometer, BD Biosciences) on the green channel for H_2_DCFDA probe or the red channel for DHE probe.

### Molecular markers analyses by western blot

Cells were harvested by trypsin treatment followed by two washes in PBS. Whole-cell extracts were prepared by resuspension of cells in lysis buffer (50 mM of Tris-HCl pH 7.5, 20 mM of NaCl, 1 mM of MgCl_2_, 0.1% SDS, 0.1% Benzonase (Novagen, Merk), cOmplet EDTA-free Protease Inhibitor Cocktail (Roche, Sigma-Aldrich) and anti-phosphatase cocktail (PhosSTO, Roche, Sigma-Aldrich) for 20 min at room temperature. Protein concentrations were determined by the Bradford method (Bio-Rad Protein assay). Samples were combined with 4X Laemmli buffer containing β-mercaptoethanol and were denatured via boiling. Proteins (30 µg) were separated by SDS-PAGE. Proteins were semi-dry transferred to nitrocellulose membranes. Blots were probed with the following antibodies: goat anti-actin (1/1000, Santa Cruz Biotechnology), rabbit anti-ERα (1/300, Santa Cruz Biotechnology) to detect oncogene induction, mouse anti-FANCD2 (1/200, Santa Cruz Biotechnology), rabbit anti-p16 (1/500, Santa Cruz Biotechnology), rabbit anti-p21 (1/500, Cell Signaling Technology), mouse anti-p53 (1/200, Santa Cruz Biotechnology), mouse anti-PCNA (1/500, Chemicon International), rabbit anti-FANCA (1/500, Bethyl Laboratories), mouse anti-vinculine (1/5000, Abcam), rabbit anti-ERK1 (1/500, Cell Signaling Technology) and goat anti-CTSL1 (1/1000, RD systems). The proteins were visualized using an enhanced chemiluminescence system (Western Bright ECL, Advansta). All Western blot quantifications were performed with the ImageJ software using densitometry measures obtained from ImageQuant LAS 4000 (GE Healthcare Life Sciences).

### Cell transfection with siRNA or plasmids

Cells were transfected at 30–50% confluence with 20 nM of siRNA and INTERFERin (Polyplus-transfection) 2 days before oncogene induction Wi-38-hTERT/ER:GFP:RAF1 or twice (3 days before oncogene induction and the day of oncogene induction) for IMR90-hTERT/ER:RAS. The following sequences were used: siFANCD2#1 (siD2 in Figures) 5′-GGAGAUUGAUGGUCUACUA-3′, siFANCD2#2 (siD2 #2 in Figures) 5′-AACAGCCAUGGAUACACUUGATT-3′ and siLacZ as control (5′-CGUCGACGGAAUAACUUCGA-3′). The transfection procedure was performed in 6-well plates according to the manufacturer’s instructions.

IMR90-hTERT/ER:RAS were transfected with 1 µg of pFLAG-FANCD2, gave by A. Constantinou’s lab (Montpellier, France) using 2 µl of TurboFect^TM^ Transfection Reagent (Thermo Fisher Scientific) 2 days before oncogene induction. Control cells were transfected with empty plasmid (pcDNA3.1). The transfection procedure was performed in Petri dish 60 mm according to the manufacturer’s instructions.

### qRT-PCR

RNA was extracted using the Maxwell® DSC simply RNA cell kit (Promega). Reverse transcription was perfomed using a RevertAid Frist Strand cDNA Synthesis Kit (Thermo Fisher Scientific) and qPCR using Maxima SYBR Green/ROX qPCR Master Mix (Thermo Fisher Scientific) on an BioRad CFX96^TM^ Real-Time System. FANCD2 was assessed using cyclofilin B as reporter gene. Primers sequences are: F: 5′-CCATGGTCACAGCACCAATA-3′, R: 5′-TCAGCACACTGGCATTTAGC-3′ for FANCD2 and F: 5′-GTGAGCGCTTCCCCGATGAG-3′, R: 5′-TGCCAAACACCACATGCTT-3′ for cyclofilin B.

### Drugs treatment

To determine the role of ROS in different processes, N-acetyl-cystein (NAC) was added in the culture media at a final concentration of 2.5 mM in HEPES (17 mM) and NaOH (130 mM) at day 0; the media was changed every day and was maintained during OIS kinetics. For some experiments, a cathepsin L inhibitor (Z-FF-FMK, Calbiochem) was added at 2.5 µM in the culture media at day 0 and maintained during OIS kinetics. Media were changed every two days.

To determine the role of the protesomal degradation of FANCD2, cells were treated with 2 µM of MG132 (Sigma) overnight.

### Statistical analysis

Analyses were performed comparing the means of two groups, with each mean obtained from at least three independent experiments. We used a paired *t* test realized by GraphPad prism software.

## Supplementary information

Supplementary informations
