# Supplementary material for: Fanconi anemia proteins counteract the implementation of the oncogene-induced senescence program
Source: Sci Rep. 2019 Nov 19;9:17024. doi: 10.1038/s41598-019-53502-w (PMC6863893; doi:10.1038/s41598-019-53502-w)
Supplement: Supplementary file 1 — Supplementary informations [file 41598_2019_53502_MOESM1_ESM.pdf]

Fanconi anemia proteins counteract the implementation of the oncogene-induced senescence program

Anne HELBLING-LECLERC[1,2,3], Françoise DESSARPS-FREICHEY[1,2,3], Caroline EVRARD[1,2,3] and Filippo ROSSELLI[1,2,3]

[1]UMR8200-CNRS, Gustave Roussy, Villejuif Cedex, France

[2]Université Paris-Sud, Université Paris-Saclay, Orsay, France

[3]Equipe labellisée "La Ligue Contre le Cancer".

Correspondence:

Anne Helbling-Leclerc and Filippo Rosselli, UMR 8200 - CNRS - Gustave Roussy, PR2, 114 rue Edouard VAILLANT, 94805 VILLEJUIF Cedex;

phone: +33(0)41.11.51.16; fax:+33 (0)1.42.11.52.44

Email: anne.helbling-leclerc@gustaveroussy.fr & filippo.rosselli@gustaveroussy.fr

SUPPLEMENTAL DATA LEGENDS

S1A and S1B. Example of cell cycle profiles obtained by FACS analysis of BrdU and PI stained IMR90* and WI38* cells.

S2A. Representative Western blots showing the time course expression of indicated protein in WI38*-oncogene activated cells proficient or deficient in FANCD2.

S2B. Quantitative analysis of the expression of the proteins analyzed as in S2A. Bar represent the mean +/_ SEM of at least 3 independent experiments. *p<0.05.

S2C. Quantitative analysis of FANCD2 expression at the indicated time point in oncogene-activated WI38* cells. Bars represent the mean +/- SEM of at least 3 independent experiments. *p< 0.05; **p<0.01.

S2D. Graphics present the time course expression of each indicated proteins relative in presence or absence of FANCD2 in IMR90* or WI38*-oncogne activated cells. Each point is mean +/- SEM of at least 3 independent experiments.

S1A

IMR90* cells

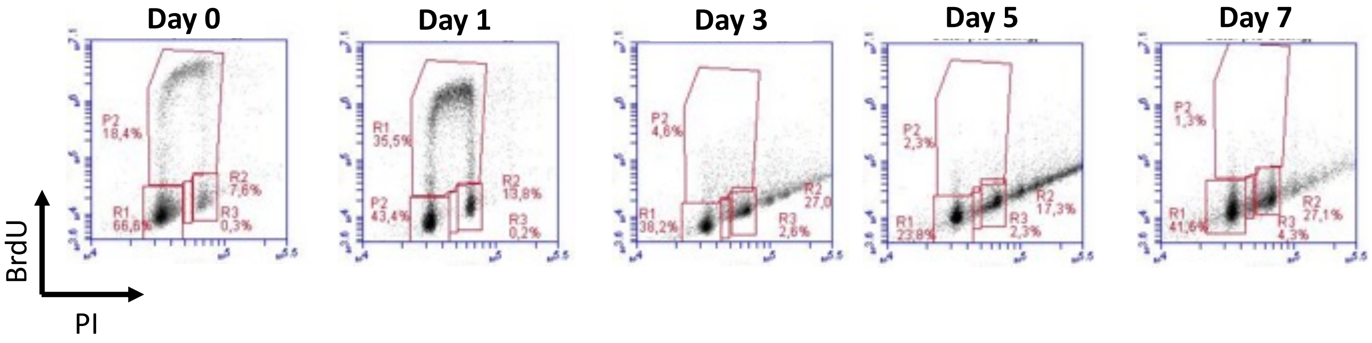

S1B

WI38* cells

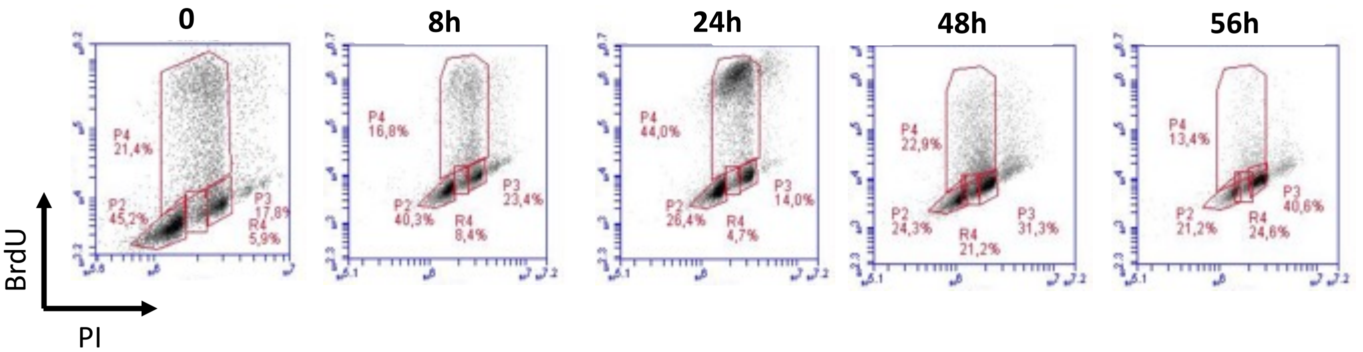

Supplemental Figure 1. Helbling-Leclerc et al.

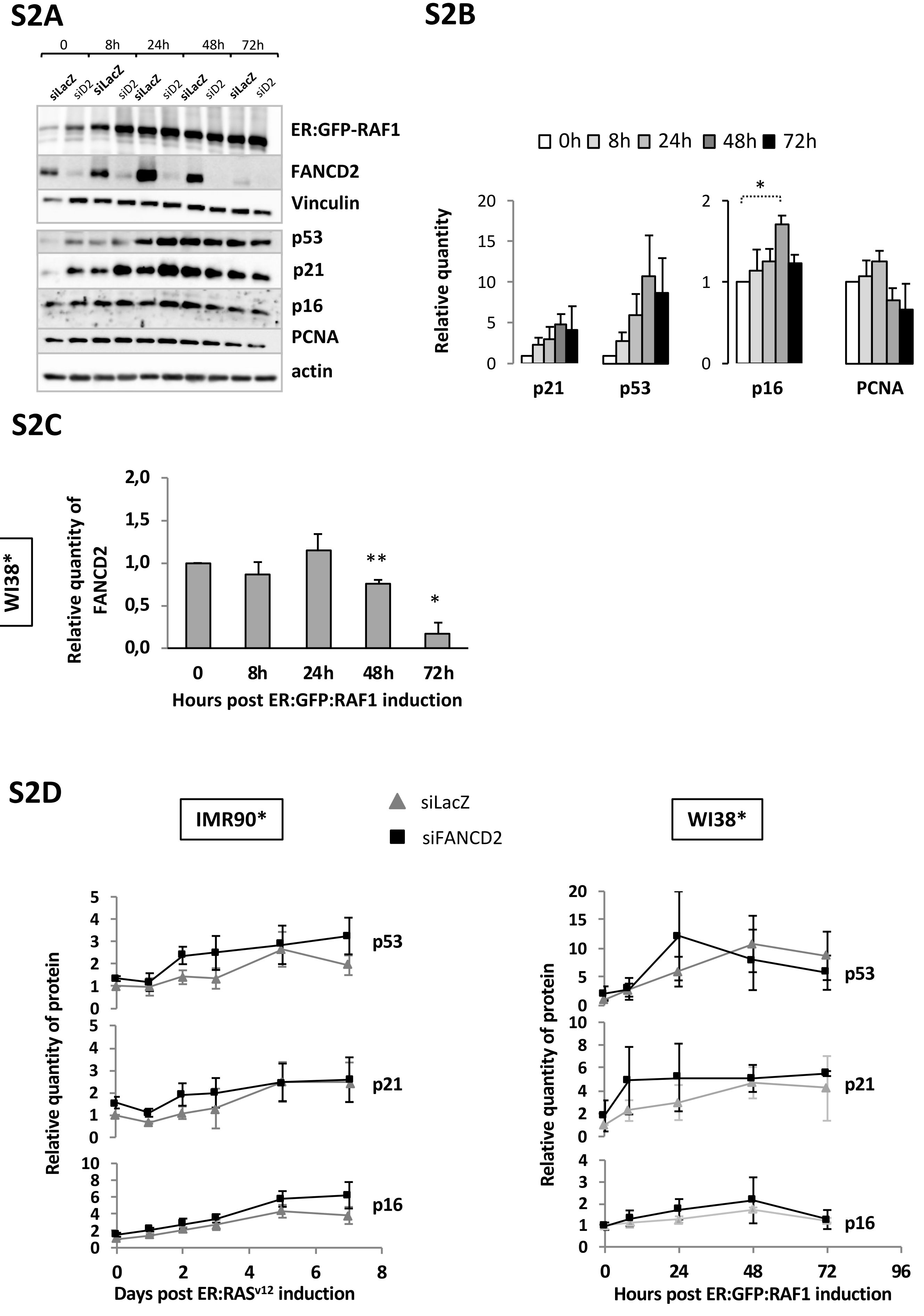

Supplemental Figure 2. Helbling-Leclerc et al.
